# A case of chondromesenchymal hamartoma of the skull base

**DOI:** 10.1016/j.radcr.2026.03.069

**Published:** 2026-05-07

**Authors:** Tatsushi Oura, Taro Shimono, Hiroki Morisako, Takeo Goto, Sayaka Tanaka, Kenichi Kohashi, Kentaro Kotani, Satoshi Oue, Ayako Omori, Hiroyuki Maeda, Shu Matsushita, Daisuke Horiuchi, Yukio Miki

**Affiliations:** a Department of Diagnostic and Interventional Radiology, Graduate School of Medicine, Osaka Metropolitan University, 1-4-3 Asahimachi, Abeno-ku, Osaka 545-8585, Japan; b Department of Neurosurgery, Graduate School of Medicine, Osaka Metropolitan University, 1-4-3 Asahimachi, Abeno-ku, Osaka 545-8585, Japan; c Department of Pathology, Graduate School of Medicine, Osaka Metropolitan University, 1-4-3 Asahimachi, Abeno-ku, Osaka 545-8585, Japan

**Keywords:** Chondromesenchymal hamartoma, Skull base, CT, MRI

## Abstract

Here, we present an unusual case of chondromesenchymal hamartoma limited to the skull base without sinonasal involvement. A 19-year-old man presented with a two-month history of diplopia and right abducens palsy. Imaging revealed a clival mass extending into the right cavernous sinus with central and peripheral calcifications, marked T2 hyperintensity, no restricted diffusion, and a heterogeneous and predominantly peripheral enhancement. Endoscopic endonasal resection was performed, and histopathological examination confirmed a chondromesenchymal hamartoma. The imaging features of chondromesenchymal hamartomas are similar to those of chondrosarcomas, making differentiation difficult. Although germline or somatic DICER1 testing was not performed in our case, the detection of DICER1 gene mutations may help establish a diagnosis.

## Introduction

Chondromesenchymal hamartoma is a rare benign tumor that commonly occurs in infants and young children. It typically arises in the chest wall and the head and neck region, most frequently involving the paranasal sinuses [1]. However, to the best of our knowledge, chondromesenchymal hamartomas limited to the skull base have not been reported previously. Herein, we present the first case of chondromesenchymal hamartoma of the skull base and describe its multimodal imaging findings.

## Case report

A 19-year-old man presented with a two-month history of diplopia. Physical examination revealed limited right eye abduction and right abducens nerve palsy. The patient had no relevant medical or family history.

**Fig. 1:**
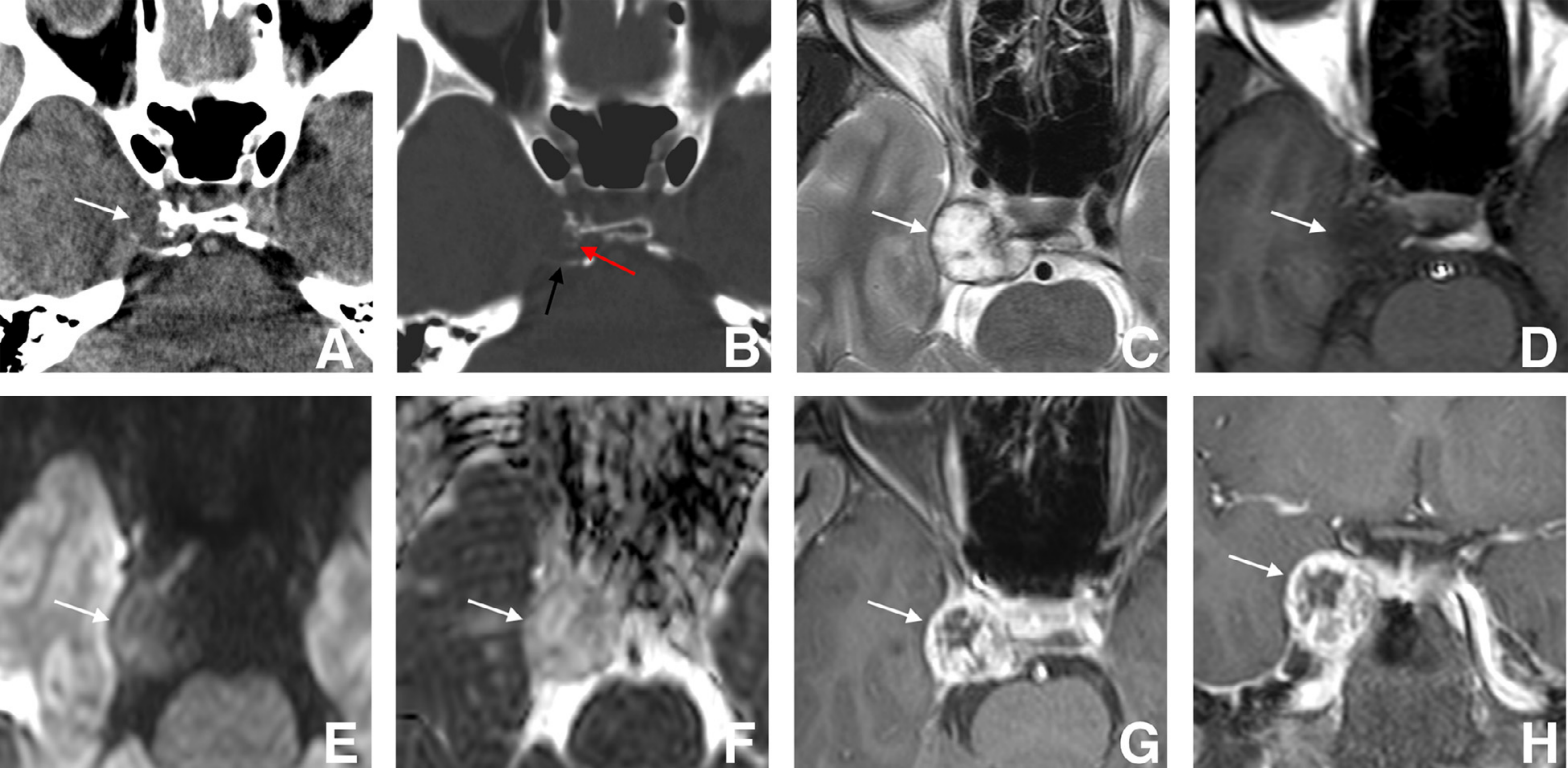
Radiological findings. (A) Axial unenhanced CT (soft tissue window) showing a low-density mass (white arrow) centered on the right clivus and extending into the right cavernous sinus with adjacent osseous destruction. (B) Axial unenhanced CT (bone window) showing central (black arrow) and peripheral (red arrow) linear calcifications. (C) Axial T2-weighted image showing a markedly hyperintense mass (white arrow). (D) Axial T1-weighted image showing a hypointense mass (white arrow). (E) Axial diffusion-weighted image (b = 1000 s/mm²) showing a hypointense mass (white arrow). (F) Axial apparent diffusion coefficient (ADC) map showing a high ADC value (1.96 × 10⁻³ mm²/s) (white arrow). (G, H) Axial (G) and coronal (H) fat-suppressed contrast-enhanced T1-weighted images showing heterogeneous and predominantly peripheral enhancement (white arrow).

Unenhanced computed tomography (CT) (Figs. 1A and B) revealed a low-density mass centered on the right clivus extending into the right cavernous sinus, with adjacent osseous destruction and central and peripheral linear calcifications. On Magnetic resonance imaging (MRI) (Figs. 1C-H), the mass appeared markedly hyperintense on T2-weighted images, hypointense on T1-weighted images, hypointense on diffusion-weighted images (b = 1000 s/mm²) and showed a high value on the apparent diffusion coefficient (ADC) map (1.96 × 10⁻³ mm²/s). After gadolinium administration, the lesion exhibited heterogeneous and predominantly peripheral enhancement.

Based on the location of the lesion and radiological findings, cartilaginous neoplasms, particularly chondrosarcomas and chordomas, were included in the differential diagnoses.

The patient underwent endoscopic endonasal tumor resection via a transmaxillary transpterygoid approach. Intraoperative findings revealed a firm mass extending from the right posterior clinoid process to the clivus and right cavernous sinus without involvement of the paranasal sinuses.

**Fig. 2:**
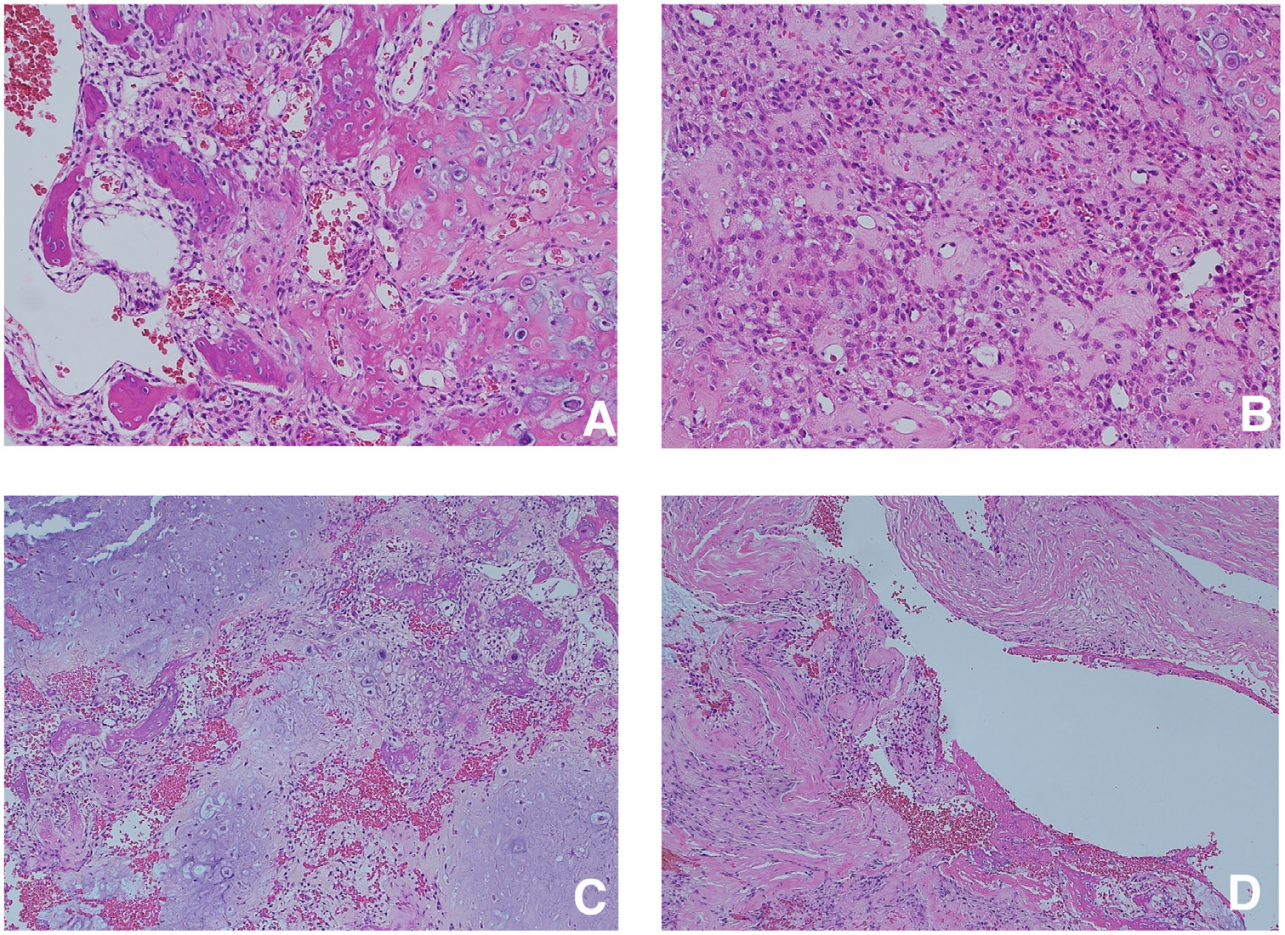
Histopathological findings (hematoxylin and eosin staining). (A) Cartilage islands showing a transition to bone trabeculae (× 400). (B) Proliferation of chondroblasts and immature mesenchymal cells (× 400). (C) Lobulated cartilage islands were observed. However, osteoclast-type giant cells were not observed (× 200). (D) Cystic spaces with partial hyalinization and internal fibrin deposition, suggestive of secondary aneurysmal bone cyst-like changes (× 200).

Histopathological examination revealed cartilage islands showing a transition to bone trabeculae and the proliferation of chondroblasts and immature mesenchymal cells (Figs. 2A, B, and C). Osteoclast-type giant cells were not observed. Cysts with partial hyalinization were observed, accompanied by internal fibrin deposition, suggesting secondary aneurysmal bone cyst-like changes (Fig. 2D). The Ki-67 labeling index was 12%.

Immunohistochemically, the cells were positive for S-100 but negative for EMA, Brachyury, and MDM2. Based on these pathological findings, a chondromesenchymal hamartoma was diagnosed. Postoperatively, the patient experienced a transient arginine vasopressin deficiency (diabetes insipidus), which subsequently resolved. No other adverse events were reported.

## Discussion

Chondromesenchymal hamartoma is a rare benign tumor first described by McDermott et al. [2]. Although the mean age at diagnosis is 5.1 years, indicating a predilection for young children, it can occur in a wide age range [1]. Chondromesenchymal hamartomas are associated with DICER1 mutations [3]. In our case, germline or somatic DICER1 testing was not performed, and there was no medical history or clinical findings suggestive of a DICER1-associated neoplasm.

In the head and neck region, it most frequently arises in the paranasal sinuses or nasal cavity and can extend into the orbit, skull base, or intracranially [1,[4], [5], [6]]. A comprehensive search of the PubMed and Google Scholar databases (through December 2025) using the keywords “chondromesenchymal hamartoma” and “skull base” yielded several reports of sinonasal CMH with secondary intracranial or skull base extension [2,[7], [8], [9], [10]]. However, to the best of our knowledge, there have been no reports of chondromesenchymal hamartoma limited to the skull base without involvement of the nasal cavity or paranasal sinuses, as observed in our case.

Radiologically, chondromesenchymal hamartoma typically demonstrates internal calcifications on CT, along with adjacent bone remodeling or destruction. On MRI, the lesion usually shows high signal intensity on T2-weighted images (although areas of low signal may be observed if there is an internal hemorrhage) and a low-to-isointense signal on T1-weighted images. Cystic components are observed in approximately 40% of cases. Diffusion restriction is not usually observed, and lesions often exhibit heterogeneous and predominantly peripheral contrast enhancement [4]. Although no cystic component was identified in the present case, the other radiological findings were consistent with those previously reported for chondromesenchymal hamartomas.

The primary differential diagnoses included chondrosarcoma and chordoma. In the present case, the mass was limited to the skull base without sinonasal involvement, and differentiation from skull base tumors was particularly challenging. Chordomas display imaging features similar to those of the present case, including high signal intensity on T2-weighted images and heterogeneous contrast enhancement [11,12]. However, chordomas typically arise at the midline of the skull base and show mildly elevated ADC values [12,13]. Furthermore, calcifications in chordomas typically represent entrapped bone fragments resulting from bone destruction and differ morphologically from those observed in the present case [14], which may serve as a useful distinguishing feature.

Chondrosarcomas, as in the present case, predominantly arise off-midline at the skull base and demonstrate high signal intensity on T2-weighted images, low signal intensity on T1-weighted images, heterogeneous contrast enhancement, and high ADC values, making differentiation particularly difficult [12,13,15]. Calcifications in chondrosarcomas reflect endochondral ossification and classically appear as ring-and-arc or popcorn-like patterns [15,16]. Although linear calcifications with a ring-and-arc-like appearance were present in our case, the absence of popcorn-like calcifications may help differentiate it from a chondrosarcoma, although prospective differentiation remains difficult. Furthermore, while CMH has a mean age at diagnosis of 5.1 years [1], the patient in the present case was 19 years old, which is atypical for CMH but considerably younger than the typical age for chondrosarcoma which most commonly occurs in the fourth decade of life [17], and the patient's age was intermediate between the two entities. Overall, the overlapping imaging findings and the patient's intermediate age rendered radiological and clinical differentiation between these entities considerably difficult**.** Although DICER1 genetic testing was not performed in the present case, it may serve as a supplementary diagnostic tool in challenging cases where the diagnosis remains uncertain and could guide long-term follow-up management.

## Conclusion

To the best of our knowledge, this is the first reported instance of a chondromesenchymal hamartoma limited to the skull base without sinonasal involvement. Radiological findings overlap with those of other skull base tumors, including chondrosarcoma and chordoma, making radiological diagnosis challenging, although the morphology of the internal calcifications may help distinguish these entities. In such cases, DICER1 genetic testing may serve as a supplementary tool to support diagnosis.

## Declaration of generative AI and AI-assisted technologies in the manuscript preparation process

During the preparation of this work, the authors used Gemini, which is based on the Gemini 3.0 Pro architecture, to improve the readability and language of the manuscript. After using this service, the authors reviewed and edited the content as needed and take full responsibility for the content of the published article.

## Data statement

The data supporting the findings of this study are available from the corresponding author, T. O. upon reasonable request.

## Patient consent

Complete written informed consent was obtained from the patient for the publication of this study and the accompanying images.
